# The Increasing Rate of Secondary Amputation in Popliteal Arterial Injury Associated with Multi-Organ Injuries and Hypotension

**Published:** 2012-12-15

**Authors:** Farooq Ahmad Ganie, Hafeezulla Lone, Mohd Lateef Wani, Nasir-u-din Wani, Abdual Ganie Ahangar, Shabir Ahmad Ganie

**Affiliations:** 1 Department of Cardiovascular and Thoracic Surgery1 SKIMS, Soura, Kashmir, India; 2 Department of General Surgery, Soura, Kashmir, India

**Keywords:** Vascular Injuries, Amputation, Thrombosis, Hypovolumic Shock

## Abstract

**Obejectives:**

To study the role of hypotension and associated injuries in increasing the chances of secondary amputation in lower limb with vascular injuries.

**Methods:**

This study was conducted in the Department of cardiovascular and thoracic surgery( CVTS ), Sher-i- Kashmir Institute of Medical Sciences, ( SKIMS ) Srinagar Kashmir India and comprised all patients sustaining vascular injury due to different causes like road traffic accident, fire arm and blast injuries or falling from height during the last five years. Following admission to our Department, the patients were divided into two groups. The first group with associated injuries was hemodynamically unstable during vascular repair or in post-operative period and the second group had no associated injuries and was hemodynamically stable during vascular repair and in post-operative period.

**Results:**

During the past five years, 95 patients were operated for lower limb vascular injury in our department. Of these 25 patients had associated multi-organ injuries and were hemodynamically unstable and needed intensive care monitoring after surgical intervention. Additionally, 10 patients died due to associated multiple organ injuries, 10 needed amputation due to recurrent thrombosis of their anastomosis, and in five patients limb salvage was achieved. Seventy patients who had isolated limb vascular injuries with no associated injuries or hypotension were hemodynamically stable and were kept in low dependency unit after vascular repair. Only Four patients from this group needed amputation for thrombosis of the anastomosis.

**Conculsion:**

Patients with shock and related injuries face significant rate of amputation. These patients whether with multi-organ injuries or isolated vascular injuries need judicious treatment for hypovolumic shock during surgical intervention and in post-operative period.

## 1. Introduction

Injury to the popliteal vessels has been recognized as one of the most limb-threatening peripheral vascular injuries so far as vascular trauma has been studied. The popliteal artery is a true end artery with a tenuous collateral supply. The popliteal vein provides the bulk of lower limb and foot drainage. This explains why injury to these vessels is so dangerous. Management of these multiply injured patients can be very challenging and restoring perfusion to an ischemic extremity needs to be accomplished within 6–8 hours, if limb is to be salvaged. Clean and sharp cut in the artery closed by primary repair behave differently than an arterial repair with RSV(Reverse saphenous vein graft) graft which needs multiple Fogarty catheterization for retrieval of thrombus. Extensive tissue damage increases chances of compartment syndrome (CS). Post–operative infection and CS are most important causes for amputation. Conventionally there are hard and soft signs of vascular injury. Hard signs which warrant immediate intervention include i) external arterial bleeding, ii) rapidly expanding hematoma, iii) palpable thrill, and audible bruit, iv) signs of acute limb ischemia. Soft signs of vascular injury warrant urgent evaluation in the form color Doppler/angiography. The successful management of vascular injury includes two goals: i) treating the life-threatening problems by fluid resuscitation, control of bleeding and ensuring adequate oxygenation; ii) repairing the injured vessel and save the limb. Arterial repairs include: reverse saphenous vein graft, primary repair, PTFE( polytetrafluroethylene) graft, ligation, and vein patch. CS leads to tissue necrosis, permanent functional impairment, or severe renal failure and death. The general consensus is that intra-compartmental pressures (ICPs) greater than 30mm Hg require intervention. During the first and second world wars, important knowledge had been gained both in diagnosis and treatment of vascular injuries, but vascular reconstructive methods were mainly introduced during the Korean and Vietnam wars with tremendous progress (1, 2).

## 2. Materials and Methods

The studied population included patients with sustained vascular injury due to different causes like road traffic accident, fire arm and blast injuries or falling from height and admitted to our center during the past five years. The patients were divided into two groups of hemodynamically unstable and stable patients during vascular rep air or in post-operative period and notes were taken of their other associated injuries. Severity of injury was assessed by MESS (mangled extremity severity score). All hemodynamically unstable patients during and after surgery, had multiple organ injury and needed intensive care monitoring. However, hemodynamically stable patients were managed in a low dependency unit after vascular repair, and had predominantly isolated lower extremity injury or associated minor injuries. All patients operated for lower limb vascular injury were followed for post-operative outcome with respect to patency of the anastomosis and adequate blood flow for viability of the limb. Distal blood flow was assessed by clinical examination followed by Doppler study to confirm doubtful clinical examination. All patients who needed primary or secondary amputation with MESS score equal to or greater than 7 were excluded from the study. The study included only patients with MESS score of <6 and confirmed to have good vascularity on table by clinical as well as by vascular Doppler examination. Hemodynamic parameters and blood gas analysis were checked during and after operation. Post-operative blood gases and electrolytes were determined four hourly in patients who needed intensive care monitoring. Other associated injuries were taken care of by respective departments including general surgery, plastic surgery, neurosurgery and by critical care specialists.

### 2.1. Operation technique and intra-operative hemodynamic parameters

After initial resuscitation and assessment, patients with confirmed arterial injury were operated under general anesthesia immediately following basic work-up. Patients with multi-organ injury were directly shifted to the tertiary care hospital from the spot of injury. Forty patients with isolated limb injury were referred after skeletal fixation by an orthopedist. Exposure was made through an incision in the aspect of knee, approximately central to site of injury. After controlling the affected arterial segment, medial proximal and distal embolectomy was performed, and backflow and inflow were assessed. Arterial repair was done either by primary end-to-end anastomosis or by reverse sephanous vein (RSV) graft. RSV was always taken from contralateral large saphenous vein. Suture material used for anastomosis was always 6^0^ double arm prolene. As for associated injuries, the patients referred to other specialties. Hemodynamic parameters, blood gas and electrolytes were checked during the operation.

## 3. Results

During five past years, 95 patients, 87 males and 8 females, aged from 18 to 45 years were operated in our department, for lower limb vascular injury. Younger patients sustained vascular injuries mostly due to motorcycle and fire arm accidents, while older patients received vascular injuries either due to blast injury or fall from height. Following surgical intervention, 25 patients with associated multi-organ injuries needed intensive care monitoring. Of these, 10 patients died due to associated multiple organ injuries, and 10 had amputation due to recurrent thrombosis of their anastomosis, and in five patients limb salvage was achieved, but their functional outcome was not as good as those who never had hypotension during surgical intervention or in post-operative period. After vascular repair, 70 patients with isolated limb vascular damage and no associated injuries were kept in low dependency unit. Patients with isolated limb vascular injury and managed in low dependency unit after surgical intervention never had prolonged hypotension, either during vascular repair or in post-operative period and four patients from this group needed amputation for thrombosis of the anastomosis. One of these patients died due to infectious complications followed by DIC even after amputation. In remaining three, one patient had atherosclerosis, one had hypercoagulabe state for protein C deficiency, and the third patient had no apparent sign of thrombosis of anastomosis.

## 4. Discussion

Advances in hemorrhage control, wound care, orthopedic and vascular surgery during Iraq and Afghanistan war have allowed greater attention to shift toward limb salvage efforts. They also suggest that hypotension may result in muscle damage at even lower compartment pressures (3). Patients with low blood pressure suffer irreversible injury at lower absolute tissue pressure than normotensive subjects. Therefore, poly-trauma patients are at increased risk of compartment syndrome (CS) from associated hypotension (4). In our study significant secondary amputation rate was found in patients of lower limb injury with associated injuries and hypotension with or without compartment syndrome in first 6 to 10 hours .According to Andrikopoulos and colleagues (5)the amputation rate can be as high as 78%. On the other hand, Razmadze and colleagues (6) reported a limb salvage rate of 77.7%. We had amputation rate of about 66.67% in patients of multi-organ injury with significant hypotension during surgical intervention and/or post-operative period, whereas an amputation rate of 5.7% was associated with isolated limb injury and no hypotension during the same period. The four significant criteria of The Mangled Extremity Severity Score (MESS), with increasing points for worsening prognosis, were skeletal/soft-tissue injury, limb ischemia, shock, and patient’s age. A MESS score of greater than or equal to 7 had a 100% predictable value for amputation (7). Our selected group of patients had a MESS score of less than 6. Bryan et al. reported that there was no correlation between delayed surgery and amputation rate in the first 24 hours. They underlined in their series that the most important determinant for amputation was the extent of soft tissue injury (8). We observed that soft tissue injury increased amputation rate, which was exponentially deteriorated by simultaneous hypotension and associated injuries. Popliteal artery trauma results in amputation more often than any other arterial injury, with 22% to 30% limb loss (9,10). Overall amputation rate in our series was 16.5%. The risk factors for amputation include occluded grafts, combined above and below knee injuries, tense compartments, arterial transections, and associated compound fractures (11). These factors increase the chances of hypotension as well as hypotension associated amputation rate. Time lags and ongoing resuscitation needs remained the ultimate Achilles heel of successful vascular surgery during the Korean War (12). Developing an early advanced resuscitation strategy for vascular trauma patients is a key component for successful limb salvage in massively injured casualties (13). Emergency War Surgery textbook strongly recommends extremity amputation in situations where a poor physiologic condition may preclude a safe vascular reconstruction (13). We also concluded that resuscitation for optimal hemodynamics and in physiological range blood gas parameters is must for successful vascular repair and limb salvage, as we had a better outcome in our patients who were hemodynamically stable with no associated injuries. In-hospital occlusion of an arterial repair is almost always related to delayed presentation or belated diagnosis of the injury, a technical mishap in the operating room, and occlusion of the venous outflow from the area of injury. In a patient with delayed presentation or diagnosis, in situ distal arterial thrombosis may occur within 6 hours (14). Both delayed presentation and diagnosis are usually associated with serious clinical status and critical hemodynamic condition of patient, which damage the vascular patency and increase the primary as well as secondary amputation rate. The amputation rate, mostly secondary, in lower limb injuries was 37% (15). Secondary amputation is significantly preventable by maintaining systolic blood pressure above 100 mm Hg by fluid resuscitation, control of sepsis and if needed ionotropic support along with optimal anticoagulation, healthy wound care and early fasciotomy as needed. Transfusion of more than 10 U of packed cells due to nearby or remote injury bleeding was negatively associated with limb salvage rate (15). Optimal resuscitation for bleeding improves both secondary and primary amputation rates. Patients with injuries and presenting with frank ischemia or active hemorrhage and shock have a poor prognosis and demand more urgent management than those with lesser injuries in which perfusion remains intact. Shock and crush injuries are associated with a significant rate of amputation (15) and significantly deteriorate the outcome of vascular repair. Primary amputation is determined at first sight, but a patient who has undergone primary vascular repair is deemed to have a salvageable limb. Loss of this re-vascularised limb is preventable by maintaining optimal systolic blood pressure along with other physiological parameters. Although surgical technique affects outcome, results are primarily dependent on early detection of vascular injury followed by immediate treatment (16). Optimal time and technique for vascular repair should be accompanied by optimal resuscitation for physiologically acceptable hemodynamics. The primary interventions that may improve limb salvage include liberal application of fasciotomy as well as early use of anticoagulation (17). Optimal technique, primary liberal fasciotomy and optimal use of anticoagulants are primary determinants of successful vascular repair, but concurrent monitoring of hemodynamic parameters affects anastomotic patency to a significant extent. The principles of rapid evacuation, temporary shunting, and early reconstruction affect early and satisfactory in-theater limb salvage (18). Early thrombosis rate was 6.4%, which is in the reported range. Anastomotic narrowing, poor arterial coaptation, adventitial inversion, extensive soft tissue injury and delayed revascularization of the traumatized limb are the probable factors responsible for early thrombosis in these patients, and if avoided can reduce the thrombosis of arterial reconstruction (19). All these factors were seen to have complementary role in hypotension with increasing chances of secondary amputation. A major risk factor for limb loss is the development of compartment syndrome in one or more osteofascial compartments rises (20). Hypotension and compartment syndrome were observed to cause vicious cycle for thrombosis of the anastomosis. Hemorrhagic shock exacerbates the negative impact of extremity ischemia and reducing the ischemic threshold of the limb to less than 3 hours; under these conditions restoration of perfusion within 1 hour is necessary to achieve neuromuscular recovery (21, 22). Amputation was not performed in five patients with favorable physiological hemodynamic parameters and blood gases, who needed intensive care monitoring for associated multi-organ injuries. Severe soft tissue injury, concomitant venous injuries, fractures, shock and a crushed extremity were found to be significantly associated with amputation (23-25). The rate of amputation decreases with shock therapy, use of antibiotics, better bone stabilization by external fixators, treatment of bone defects by internal bone transport, soft tissue reconstruction such as local and free flap and rapid transportation (26).

Patients with vascular and multi organ injuries ,active hemorrhage and shock have a poor prognosis and demand more urgent management than do patients with isolated limb injuries in which perfusion and blood pressure remains optimal. Shock is associated with a significant rate of amputation. These patients, whether with multi-organ or isolated vascular injuries, need judicious treatment for hypovolumic shock during surgical intervention and post-operative period.
